# Feature Down-Selection to Improve Supervised Classification by Machine Learning on Mass Spectrometry Imaging Data

**DOI:** 10.3390/molecules31122077

**Published:** 2026-06-13

**Authors:** Braysen Miller, Aleesa E. Chua, Madeline Isom, Eden P. Go, Emily R. Sekera, Amanda B. Hummon, Heather Desaire

**Affiliations:** 1 Department of Chemistry, University of Kansas, 1450 Jayhawk Blvd, Lawrence, KS 66045, USA; braysenmiller@ku.edu (B.M.);; 2 Department of Chemistry and Biochemistry, The Ohio State University, 281 W Lane Ave, Columbus, OH 43210, USA

**Keywords:** mass spectrometry, mass spectrometry imaging, machine learning, feature selection, lipids, spheroids, data analysis

## Abstract

The advancements made in the mass spectrometry imaging (MSI) field have allowed for the generation of very large-scale data sets. These data are often interrogated by machine learning (ML), although storing and handling data sets of this size can be difficult. To aid impacted researchers, we seek to evaluate feature reduction strategies that will minimize the amount of data stored while still maintaining the ability to correctly classify the data. Two different feature selection strategies are tested on six different data sets, leveraging XGBoost as the machine learning algorithm. The study provides evidence that selecting features based on the greatest average abundance across all samples is best suited to scale down the feature set at a more modest trimming level, while selecting features based on statistical analysis via a Student’s *t*-test is better suited for a more aggressive trimming level. These trends were present regardless of training set size or cross-validation strategy. The results from this work provide insight into when these feature filtering steps can be used effectively and when another data reduction strategy, including not restricting the data set, should be considered.

## 1. Introduction

Mass spectrometry imaging (MSI), which commonly leverages a MALDI interface, has an established foothold in many different fields from proteomics [1,2,3,4], to lipidomics [5,6,7], to drug metabolism and distribution studies [8,9,10]. This technique provides chemical information in a spatially resolved format across a variety of sample types, providing scientists with a method for label-free imaging and structural analysis of biological samples. While this technique has already influenced modern science extensively, further development of MSI methods is ongoing, and they will further push the limits of what this technology can do.

One of the most impactful developments is the increase in spatial resolution. During the late 1990s to early 2000s, still within the early days of MALDI-MSI experimentation, spatial resolution was limited with expected values of approximately 180 µm [11]. Some of the technological advances necessary for improving the resolution of MALDI-MSI for cellular or enabling routine sub-cellular imaging are: the reduction in spot size of the laser beam, reduced data acquisition times, and the improvement of data analysis software for image analysis [12]. Within the last few decades, strides have been made to push the spatial resolution below 5 µm [13,14,15]. Today even some high-performing commercially available instruments can have spot sizes as small as 5 µm [16,17,18]. MALDI-MSI methods can even extend below 5 µm resolution, typically through modifications of commercial instruments [15,19,20,21] and/or oversampling techniques, which can allow spatial resolution smaller than the laser spot size [22,23].

This improvement in spatial resolution leads to an increased quantity of pixels to process, and because of this, major developments in data acquisition times need to be established to prevent exhaustive analysis times. During the dawn of MALDI-MSI, data acquisition times of commercial instruments were 1–2 min per pixel [12]. The field has addressed this challenge and decreased pixel acquisition rates down to approximately 50 Hz with some instruments capable of rates exceeding 100 Hz [24,25,26]. These advancements have catapulted the development of MALDI-MSI methods to higher standards creating new possibilities in science.

Consequently, as the MALDI-MSI technique has benefitted from increased resolution and scanning speeds, new hurdles are created simultaneously. More specifically, the amount of data capable of being produced increases drastically. This causes problems in data processing and even data storage in some cases. Along with this, new instrument modalities can increase data set sizes. Currently, one of the most pertinent examples of this is ion mobility mass spectrometry, where additional ion mobility information is stored alongside all the mass spectral data, leading to more expensive data storage costs. The work presented addresses this problem by testing post-acquisition strategies that would allow researchers to retain useful information in their samples while drastically reducing their data set sizes. The methods we test herein are particularly suited to those leveraging supervised classification methods, one type of machine learning, on their data.

Machine learning is a mathematical approach that uses information about samples of a known type (ex: healthy and disease state) to predict the type of similar, unknown samples. For example, it could be used on MSI data to determine the difference between healthy tissue and cancerous tissue [27,28,29] or to classify the type of tissue (ex: source of metastatic tumors or distinguishing hepatocellular carcinoma from cirrhosis) [30,31]. Furthermore, it has been used to identify different Enterococci species [32]. When spatial information is not a requisite aspect of the analysis, machine learning can also be paired with other types of non-imaging mass spectrometry (MS) data for similar purposes. For example, it has been used for detection of pancreatic ductal adenocarcinoma [33], to detect impurities in pharmaceuticals [34], to determine variability in lipid profiles on different regions of the face [35], and to optimize sample storage methods for lipids extracted from latent fingerprints [36]. These use cases for combining machine learning with mass spectrometry data outside the field of MSI have similar needs for data reduction strategies.

In this work, we combine data reduction techniques and machine learning on both imaging and non-imaging mass spectrometry data. The objective of doing so is to understand the extent that MSI or non-imaging MS data files could be reduced without eliminating any information that could be useful in discerning between the sample types of interest. We tested two different data reduction methods at two differing levels of reduction, one retaining 0.03–0.09% of the data and the other retaining 3–9% of the data. Multiple data sets and classification strategies were used to determine if there is a link between the best data reduction strategy and the classification method. In general, we demonstrate that a variety of data sets can be reduced by over 99.9% in some cases without losing the ability to discriminate between sample types, using the XGBoost classifier [37]. This work provides a possible solution to the expanding need for data management due to immense data sets that are now possible to be produced by MSI and other mass spectrometry methods.

## 2. Results and Discussion

### 2.1. Overview

**Figure 1:**
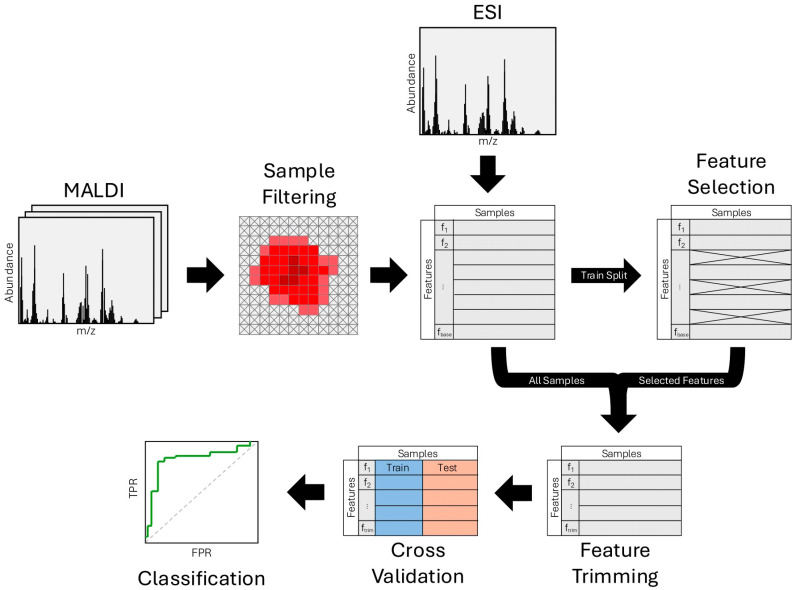
Analysis workflow. After removing empty pixels (in MALDI-MSI data), spectra are processed into matrices of samples and features. Features are selected based on training data, and they are applied to test data. Classification performance is determined by cross-validation.

Figure 1 describes the workflow for this project. It begins by filtering the raw MSI data, where only pixels with total ion counts greater than 200 are passed through to a data matrix composed of samples and features, while the non-imaging MS data is directly read into the data matrix with no filtering step. From there, the data is split into a train and test set prior to the training set being passed through different feature selection algorithms. The resulting training data is used to determine feature trimming, and both the train and the test set are trimmed using the same features prior to testing the classifiers. Throughout this work, the effects of these feature selection methods are explored using the XGBoost [37] machine learning algorithm. To interpret the impact of the feature selection techniques, classification accuracy, area under the (receiver operating characteristic) curve (AUC), DeLong tests for the AUC-ROC values, and the data set size in bytes are compared.

### 2.2. Data Sets Tested

Table 1 describes six different data sets, and their characteristics, that were used in the study. Throughout this work, two main types of data sets were employed. The first being MALDI-MSI data sets, where colon carcinoma spheroids, which had been grown for different lengths of time, were compared. The raw MALDI-MSI data is available from Zenodo.org with the following DOI: https://doi.org/10.5281/zenodo.19930148. The spheroid preparation for this data set has been described in detail in a previous work [38]. The spheroids underwent MSI analysis on day 12 or day 13 of their growth period. These data were chosen due to the expectation that the spheroids from days 12 and 13 would be highly similar to each other and therefore offer a challenging classification problem. The spheroids were analyzed in both positive and negative ion mode at both 1:9 and 9:1 train:test splits; using these parameters the single MALDI-MSI data set was split into four distinct data sets based on the parameters used, as seen in Table 1. Two additional ESI data sets were also subjected to the workflow to determine the impact of feature reduction on non-imaging MS data sets which typically are smaller in terms of overall storage cost. These two lipidomics data sets have been described in previous publications [36,39]; the larger of the two investigated the impact of sample storage conditions on sebum lipid samples [36], while the smaller looked at variation between the sebum lipid profile depending on the age of the donor [39].

### 2.3. Feature Selection Strategies

Two distinct feature selection strategies were investigated in this work, one selecting the features by feature abundance, where the sum of the normalized feature values for all samples was calculated and the k features with the greatest sums were retained. While the other selected the features based on their ability to discriminate the training data, where a Student’s *t*-test was performed on the normalized feature values between both sample classes for each feature, then the k features with the lowest *p*-values were retained. Henceforth, we refer to these as the abundance and significance methods, respectively. Down-selecting features by removing the least-abundant species is a common data preprocessing method prior to supervised classification [36,39,40,41]. However, the impact of this step on classification accuracy has not been reported to our knowledge. When employing this approach, researchers typically save features where 0.1 to 1% of the samples have nonzero values, which can result in saving as many as 80% of features in some cases [36]. Down-selecting features by *p*-value is a very common approach when proposing biomarker panels based on omics data [42,43,44,45,46]; here researchers can save less than 5% of features, although some studies have found success selecting much fewer [42]. While this approach is advantageous in drastically reducing the number of molecules needing to be analyzed to identify a disease state, it may have disadvantages for MSI experiments, when the main goal is preserving classification accuracy as much as possible, and having a larger list of features is tolerable. Thus, we sought to compare these two common feature down-selection strategies, with the goal of understanding how classification accuracy could be best preserved while reducing the file sizes of the data. Note, both feature selection methods are employed in the same way they are usually used, with modest trimming when selecting based on abundance and aggressive trimming when selecting based on significance. After this comparison, an additional study is conducted where both feature selection methods are used for a heavy level of trimming, selecting only the 10 most impactful features.

### 2.4. File Size Reduction

When assessing the effects of these feature selection methods on MS data, one of the most straightforward metrics that can be tracked is the size of the data itself. Therefore, the file size of the input matrices prior to classification was determined and used to compare the extent of reduction for each feature trimming level. These file sizes can be seen listed in Table 2, accompanied by their respective number of features and the reduction in file size from the original data sets. While the size of the original MSI data sets in this study on the order of gigabytes is manageable, the data storage cost can become expensive rapidly when storing thousands of data sets. It is also possible for individual MSI data sets to exceed storage costs far beyond those tested in this study, with some requiring over a terabyte of storage. Upon feature selection, the data storage cost for each data set was reduced by at least 90% for both feature trimming levels. While this large reduction in file size is ideal, classification accuracy must be maintained as well, or the data reduction step does not improve the workflow.

### 2.5. Method Evaluation

Using the heavily trimmed, modestly trimmed, and original data sets described in Table 2, we performed supervised classification and report the accuracy and AUC on the held-out test sets in Table 3, where the results for the XGBoost classifier are reported. In addition to the raw performance metrics for each data set, we also report the change in metric from the original data set to more clearly show the effect that each feature selection technique has on classification accuracy. It is evident that using these two different levels of feature selection, the data sets that were modestly trimmed, using the abundance feature selection method, were more similar to the original data sets than those that were heavily trimmed using the significance feature selection method.

Note: Prior to selecting the XGBoost method in Table 3, we had also completed a benchmarking study to ensure that the optimal classifier was used for these data. The Aristotle Classifier [47] and support vector machine (SVM) [48] were both compared to XGBoost. For these data sets, XGBoost generally outperformed the Aristotle classifier while performing similarly to SVM. Due to memory limitations, SVM could not run the original non-trimmed MSI data sets providing no baseline to compare with the trimmed data sets. The results for these classifiers are shown in Table S1 in the Supplementary Materials. Due to these factors, XGBoost was used to perform all assessments about the effects of feature selection herein, since a researcher would naturally choose the best-performing classifier to carry out their work.

**Figure 2:**
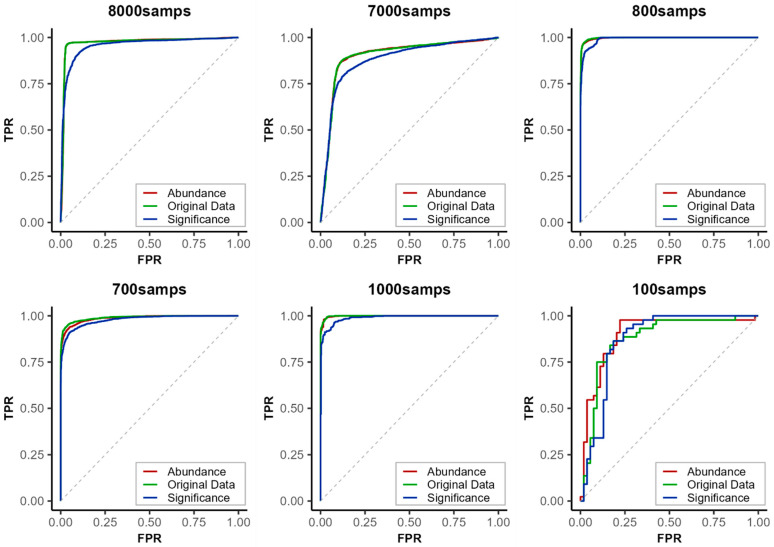
Receiver operator characteristic (ROC) curves for each data set using the XGBoost classifier. Each plot contains a curve for each feature selection method: original data, moderately trimmed by abundance, and heavily trimmed by significance. A gray reference line represents an AUC value of 0.5.

The ROC curves for each data set are shown in Figure 2, where each plot is representative of a different data set, while each line represents the original data set or one of the two feature selection methods. Each plot displayed steep curves and large AUC values for each feature selection method. For most data sets, the abundance feature selection method and the original data set ROC curves overlap very closely, while the significance feature selection method separates slightly. One noticeable outlier to this trend is the “100samps” plot where all three methods overlapped each other. This is likely due to the reduced precision from the small sample size of this data set in comparison to the other five data sets [49].

**Figure 3:**
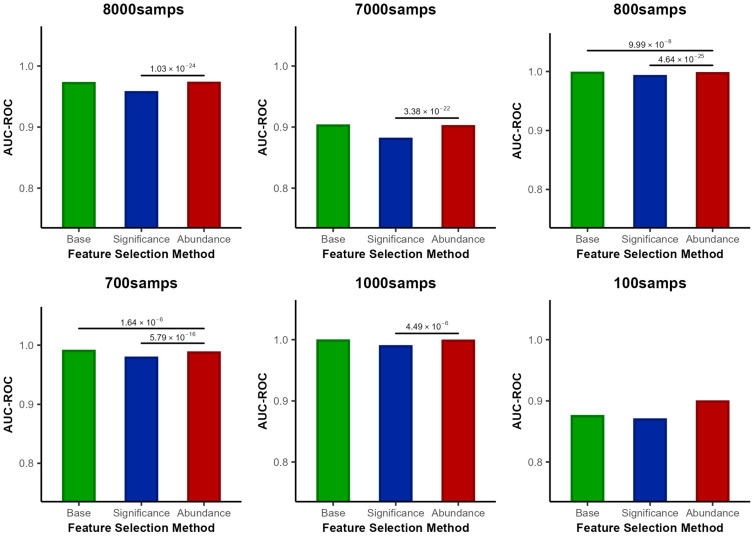
AUC values for each data set using the XGBoost classifier with different levels of trimming. Statistical significance (*p* < 0.001) is marked with the respective *p*-value. Statistically insignificant (*p* > 0.001) *p*-values are not shown.

Using the DeLong test, we also evaluated whether the differences between the AUCs for the different data sets were statistically significant, and these results are shown in Figure 3. Consistent with the initial observations of the ROC curves, there were significant differences between the AUCs for all but the “100samps” data when comparing the most heavily trimmed data set to the original data set; while, when considering the more modest trimming strategy, significant differences were only observed in the “800samps” and “700samps” data sets. The lack of statistical differences between the original and the more modestly trimmed feature set, which saved the most abundant features, suggests that the abundance feature selection method may be an optimal data reduction strategy when the primary goal is to retain high classification accuracy.

### 2.6. Method Selection at Extensive Trimming Levels

Upon completion of the initial experiment, it was evident that the abundance feature selection method at a modest level of trimming (1000 features) provided data that was typically equivalent to the original data set. While the abundance feature selection method may have overall outperformed the significance method, there are instances where a more aggressive level of trimming is required, such as reducing the size of a potential biomarker panel. Thus far, the data in this study do not provide useful information about which method would perform better when heavy trimming was an experimental requirement because only the significance method used extreme trimming. To determine the relative merit of the abundance versus significance method when heavy trimming is a prerequisite, both feature selection methods applied a heavy level of trimming, selecting the 10 most impactful features.

**Figure 4:**
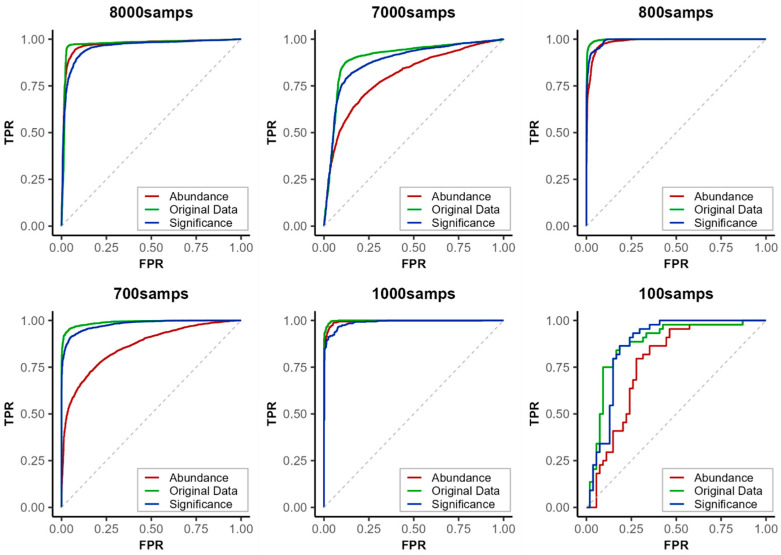
Receiver operator characteristic (ROC) curves for each data set using the XGBoost classifier. Each plot contains curves for the abundance and significance feature selection methods where both are heavily trimmed and the original data.

**Figure 5:**
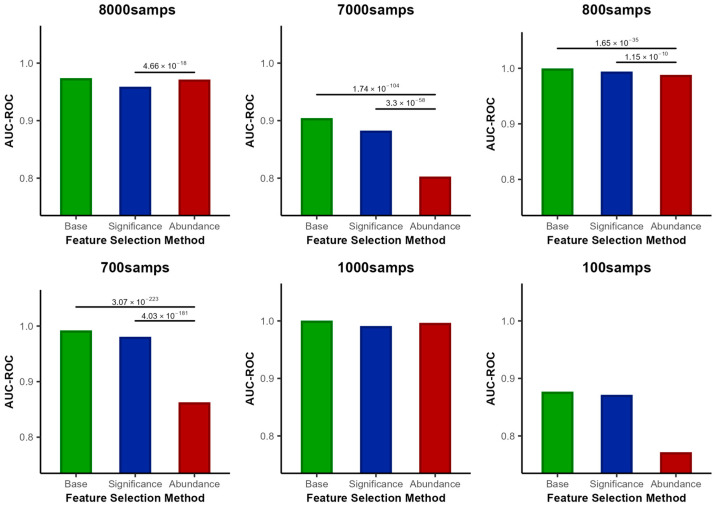
AUC values for each data set using the XGBoost classifier with equal trimming levels between the abundance and significance feature selection methods. Statistical significance (*p* < 0.001) is marked with the respective *p*-value. Statistically insignificant *p*-values (*p* > 0.001) are not shown.

The resulting ROC curves, where only the top 10 features were selected, by either abundance or significance, are shown in Figure 4. When extreme trimming is required, and only ten features are saved, the significance method outperforms the abundance feature selection method in four of the six data sets, with the “8000samps” and “1000samps” data sets being the exceptions. Statistical analysis of the AUC-ROC values was also performed through DeLong tests; the results can be seen in Figure 5. The statistical analysis reinforced the earlier results by showing that at an extreme level of trimming, the significance method performed statistically equivalent or better than the abundance feature selection method in all but the “8000samps” data set. The results from the extreme trimming tests show that the significance method is typically better when compared to the abundance method when only the top ten features are kept.

### 2.7. Experimental Trends

The results obtained herein show that the optimal feature selection method is dependent on the level of trimming used. When an extreme level of trimming is necessary, the significance feature selection method regularly outperforms the abundance method. Conversely, when a more modest level of trimming is possible, the abundance feature selection method provided results essentially equivalent to the full data set. Both of these trends were supported by statistical analysis through DeLong tests. The feature downscaling method that regularly provided results most similar to the original data set was the abundance method using a modest level of trimming. Although if a high level of trimming is required, the significance method is preferable. There was no overall impact on these trends when comparing MSI data or non-imaging MS data. Similarly, there was no effect of training set size on this trend.

### 2.8. Limitations

This study also had some limitations that could be improved upon in future studies. Specifically, the hyperparameters could be fully optimized to improve the results, but it is unlikely this will change any conclusions drawn from this work. Additionally, the test set for each data set utilized internal splitting. Using a test set composed of an external data set could help to further improve the validity of this work. Finally, more data sets could always be analyzed.

## 3. Materials and Methods

### 3.1. Spheroid Preparation and MALDI MSI Data

The feature selection methods were tested on MALDI-MSI data obtained from 10 separate spheroids grown using cells from the colon carcinoma cell line HCT 116 from each timepoint. The spheroid growth and MALDI-MSI sample preparation are described in a recent report by Fries et al. [38]. All the MSI data used in this study herein was obtained from the MALDI-MSI analysis of these spheroids. These data sets were given the classification problem of discriminating between samples harvested during day 12 or 13 of the growth period. Spheroid sections were coated with 9-aminoacridine (9AA) for analysis in both positive and negative ion mode. Both data sets were acquired on an UltrafleXtreme MALDI-TOF-TOF mass spectrometer (Bruker Daltonics, Bremen, Germany) using a smartbeam II ND:YAG 355 nm laser. The MALDI analysis was performed using a laser raster size of 25 μm in the x and y direction, with 500 shots per raster position. After data acquisition, flexImaging 4.1 software (Bruker Daltonics, Bremen, Germany) was used to transfer the MSI spectra to .imzML file format for downstream analysis using a pipeline established previously [50]. Both the positive and negative ion mode MALDI-MSI data sets were binned to contain 32,000 *m*/*z* bins; this is the reason both data sets have exactly the same number of features. The additional data sets used herein consisted of sebum lipid samples from latent fingerprints. The sample preparation and data acquisition for these data sets are described previously [36,39]. This study is a secondary analysis of these earlier-collected data sets. In these non-imaging data sets, a total of 1100 different fingerprint samples were analyzed in two different studies, which both used high-resolution direct flow injection on an Orbitrap Fusion Tribrid mass spectrometer (Thermo Scientific, San Jose, CA, USA) coupled to a Waters Acquity UPLC (Milford, MA, USA). The protocols and associated instrument parameters can be found in the original studies [36,39].

### 3.2. Data Preprocessing

#### 3.2.1. Reading the Mass Spectrum Files

All data analysis was performed in RStudio, R version 4.4.1. All raw feature data was obtained from their respective works and read into individual input matrices within RStudio. The MALDI-MSI data utilized a bin width of 0.0125 Da [40], while the ESI data used a bin width of 0.1 Da [36] or 0.01 Da [39].

#### 3.2.2. Creation of the Input Matrix

Upon extraction of the raw data matrices, the MSI data underwent a filtering step where pixels with total ion counts of less than 200 were removed from the data set; this value is about 1.5% of the maximum abundance for the imaging data sets. This was done to negate the effects of training the ML model on empty pixels where the spheroid was not present. Each of the 20 spheroids used for this study underwent analysis, resulting in a total of 20 separate raw data matrices. Each of these separate matrices was stacked to create a combined raw data matrix where each row was still representative of a specific bin of the mass spectrum, but the columns were composed of each pixel in the MSI image from all 20 individual MSI spectra, resulting in data sets with sample counts approaching 10^4^ samples. The ESI-MS raw data matrices had fewer sample counts, with the larger of the two having 1012 samples.

### 3.3. Feature Selection

Each data set in this work was exposed to three levels of feature selection, one being no selection (where all features were retained), the other methods being selection of 1000 or 10 features. There were also two separate feature selection methods tested, one retaining the most statistically significant features, while the other retained the most abundant features. Both feature selection methods were performed using a script developed in-house. The significance method selects features based on the 10 features with the lowest *p*-values obtained by performing a Student’s *t*-test for each feature between all the training samples for each class. Selecting features based on abundance was tested using both the top 1000 and 10 features with selection based on the greatest mean values across all training samples. In each experiment, only the training data was used when performing any feature selection, and results were exclusively reported on held-out test data. This was done to eliminate any potential bias of selecting features based on test data [51]. Once the selected features were determined, they were stored and the initial combined normalized data matrix was trimmed retaining only the specific features selected. The resulting matrix became the input matrix for classification.

### 3.4. Data Analysis

#### 3.4.1. Classification

The effect of each feature selection method was assessed using the XGBoost classifier [37]. XGBoost was chosen in this study due to its effectiveness at interpreting high-dimensional data and the robustness of the model. XGBoost is already a highly regarded model, established to be effective at interpreting MS data [36,39,40,52]. XGBoost is also preferred for MS data as opposed to a deep learning model, since it is simple to extract the top features and identify them based on their *m*/*z*. The following hyperparameter selection for the XGBoost classifier was used in this study: booster = “gbtree”, objective = “binary:logistic”, nrounds = 50, maximize = F. This hyperparameter setup was not optimized but found success in a previous study with a highly similar data set [40]. For each MSI data set, spheroid-level train:test splits of 1:9 and 9:1 were tested, where all the pixels for a given spheroid were clustered into either the train or the test sets. Spheroid-level splitting is necessary as splitting at a pixel-level would cause severe data leakage due to potential dependencies among MSI pixels of the same spheroid. Both non-imaging data sets utilized leave-one-out cross-validation (LOOCV).

#### 3.4.2. Classifier Benchmarking

Additional benchmarking was performed using two additional classifiers to ensure XGBoost was an effective choice, these being: the Aristotle classifier [47] and SVM [48]. For SVM classification the following hyperparameters were used: type = “C-classification”, kernel = “linear”, and probability = T. The two hyperparameters for the Aristotle classifier were set to x = 6 and repeats = 300 in this study. After testing, the results showed XGBoost was the most optimal classifier out of those tested for these data sets; therefore, all classification comparisons performed herein were done using XGBoost.

#### 3.4.3. Performance Metrics

Multiple metrics were used to evaluate the effects of the feature selection methods. Classification accuracy, defined as the percent of correctly classified samples, was the first metric tested. Alongside accuracy, the area under the receiver operator characteristic (ROC) curve was calculated. These ROC curves were created using the “pROC” package v 1.18.5 in R [53]. The ROC curve was built by plotting the true positive rate (TPR) and the false positive rate (FPR) at different decision thresholds. These thresholds were calculated by ordering the decision probabilities for all samples in increasing order and selecting the midpoint between each sequential pair of probabilities. After the ROC curve was plotted, the area under the curve (AUC) was computed. Alongside the AUC computations, DeLong tests were performed to determine any significant differences between the AUC values for each different method used [54]. The final metric of interest in this work was the relative data storage cost. This was inferred by determining the file size in bytes of the respective input matrices and was utilized in this work to track the lowered data storage cost after feature selection. Each metric was used to track the effects of these feature selection methods on both their ability to correctly classify the data and the amount of storage saved. These metrics portrayed the strengths and weaknesses of each feature selection method tested.

## 4. Conclusions

We have investigated the relationship between the extent of feature down-selection and performance of supervised classification algorithms using two distinct feature selection methods on mass spectrometry data sets. The methods “significance” and “abundance” choose features based on either the *p*-value of the individual feature or the highest mean ion intensity respectively. The performance of the XGBoost classifier was monitored using classification accuracy, AUC-ROC, and statistical analysis via the DeLong test. The results showed that overall, the abundance method was more effective at a modest level of feature selection, while the significance method generally maintained high performance even with a much more drastic level of feature trimming. These results were consistent throughout many different test cases with varying sample numbers, training set sizes, or whether the data used was MSI. Knowing the effects of these feature selection methods can help improve supervised classification workflows and help provide scientists the tools they need to make informed decisions for their methods.

## Figures and Tables

**Table 1:** Data set characteristics.

Data Set Identifier ^a^	Classification Problem	Ionization Method	Ionization Mode	Cross-Validation ^b^	Number of Samples ^c^	Number of Features
8000samps	Spheroid Age	MALDI	+	9:1	8632	32,000
7000samps	Spheroid Age	MALDI	−	9:1	7438	32,000
800samps	Spheroid Age	MALDI	+	1:9	8632	32,000
700samps	Spheroid Age	MALDI	−	1:9	7438	32,000
1000samps	Sample Storage Duration	ESI	+	LOOCV	1012	11,167
100samps	Donor Age	ESI	+	LOOCV	98	37,919

^a^ Data set identifiers are the rounded number of samples used in the training set for that data set. ^b^ Cross-validations shown are represented as train:test splits. ^c^ In the context of the MSI experiments, one sample is one pixel. For ESI experiments, one sample is one biological sample.

**Table 2:** File size reductions.

Data Set Identifier	Trimming Level	Number of Features	File Size (Bytes) ^b^	∆File Size (Bytes) ^a^	% Data Size Reduction
8000samps	None	32,000	2.210 · 10^9^		
8000samps	Heavy	10	6.908 · 10^5^	2.209 · 10^9^	99.97%
8000samps	Modest	1000	6.906 · 10^7^	2.141 · 10^9^	96.87%
7000samps	None	32,000	1.904 · 10^9^		
7000samps	Heavy	10	5.953 · 10^5^	1.904 · 10^9^	99.97%
7000samps	Modest	1000	5.950 · 10^7^	1.845 · 10^9^	96.87%
800samps	None	32,000	2.210 · 10^9^		
800samps	Heavy	10	6.908 · 10^5^	2.209 · 10^9^	99.97%
800samps	Modest	1000	6.906 · 10^7^	2.141 · 10^9^	96.87%
700samps	None	32,000	1.904 · 10^9^		
700samps	Heavy	10	5.953 · 10^5^	1.904 · 10^9^	99.97%
700samps	Modest	1000	5.950 · 10^7^	1.845 · 10^9^	96.87%
1000samps	None	11,167	9.048 · 10^7^		
1000samps	Heavy	10	1.543 · 10^5^	9.033 · 10^7^	99.83%
1000samps	Modest	1000	8.169 · 10^6^	8.231 · 10^7^	90.97%
100samps	None	37,919	2.974 · 10^7^		
100samps	Heavy	10	1.769 · 10^4^	2.972 · 10^7^	99.94%
100samps	Modest	1000	7.938 · 10^5^	2.894 · 10^7^	97.33%

^a^ File size reductions for each feature trimming level on each data set. ^b^ It should be noted that the file sizes for the 8000/800 and 7000/700 data set pairs are the same respectively. This is because both data sets use the same input file and only differ in the cross-validation step.

**Table 3:** XGBoost performance metrics.

Data Set Identifier ^a^	AUC-ROC	Accuracy	ΔAUC-ROC ^b^	ΔAccuracy ^b^
8000samps_o	0.972	96.20%		
8000samps_s	0.957	90.77%	−0.015	−5.43%
8000samps_a	0.972	96.18%	0.000	−0.02%
8000samps_a*	0.970	93.35%	−0.002	−2.85%
7000samps_o	0.902	87.48%		
7000samps_s	0.881	83.18%	−0.021	−4.30%
7000samps_a	0.901	87.60%	−0.001	+0.12%
7000samps_a*	0.801	73.60%	−0.101	−13.88%
800samps_o	0.998	97.27%		
800samps_s	0.992	94.46%	−0.006	−2.81%
800samps_a	0.997	95.68%	−0.001	−1.59%
800samps_a*	0.986	89.98%	−0.012	−7.29%
700samps_o	0.990	94.60%		
700samps_s	0.978	91.95%	−0.012	−2.65%
700samps_a	0.987	94.06%	−0.003	−0.54%
700samps_a*	0.861	77.68%	−0.129	−16.92%
1000samps_o	0.999	97.43%		
1000samps_s	0.989	93.58%	−0.010	−3.85%
1000samps_a	0.998	97.63%	−0.001	+0.20%
1000samps_a*	0.995	96.34%	−0.004	−1.09%
100samps_o	0.875	81.63%		
100samps_s	0.870	81.63%	−0.005	0.00%
100samps_a	0.899	83.67%	+0.024	+2.04%
100samps_a*	0.770	69.39%	−0.105	−12.24%

^a^ Data set identifier used to distinguish each data set where the numeric value is the rounded training samples and the letter is the feature trimming method (o = original data set, s = significance feature selection, a = modestly trimmed abundance feature selection, and a* = heavily trimmed abundance feature selection). ^b^ The delta values are the changes in the performance metric compared to the respective original data set.

## Data Availability

The raw MALDI data for this study is available at Zenodo.org with the following DOI: https://doi.org/10.5281/zenodo.19930148. The ESI data was previously made available in the Supplementary Materials of Refs. [36,39].

## Supplementary Materials

The following supporting information can be downloaded at: https://www.mdpi.com/article/10.3390/molecules31122077/s1, Table S1: Additional Classifier Performance Metrics.
