# Supplementary material for: Feature Down-Selection to Improve Supervised Classification by Machine Learning on Mass Spectrometry Imaging Data
Source: Molecules. 2026 Jun 13;31(12):2077. doi: 10.3390/molecules31122077 (PMC13304621; doi:10.3390/molecules31122077)
Supplement: Supplementary file 1 [file molecules-31-02077-s001.zip › molecules-4323021-supplementary.pdf]

Table S1. Additional Classifier Performance Metrics

	Aristotle Classifier		SVM[b]	
**Data Set Identifier[a]**	**AUC-ROC**	**Accuracy**	**AUC-ROC**	**Accuracy**
8000samps_o	0.972	93.25%		
8000samps_s	0.923	**85.75%**	0.954	90.69%
8000samps_a	0.975	94.75%	0.975	96.72%
8000samps_a*	**0.896**	**85.16%**	0.955	91.76%
7000samps_o	**0.791**	**74.82%**		
7000samps_s	0.871	80.45%	0.875	83.54%
7000samps_a	**0.791**	**75.52%**	0.889	84.57%
7000samps_a*	**0.589**	**57.56%**	**0.751**	**69.92%**
800samps_o	0.996	93.83%		
800samps_s	0.985	**89.64%**	0.995	95.37%
800samps_a	0.996	93.89%	0.999	99.11%
800samps_a*	**0.930**	**84.73%**	0.989	95.78%
700samps_o	**0.873**	**81.41%**		
700samps_s	0.964	87.54%	0.972	92.15%
700samps_a	**0.877**	**82.16%**	0.983	93.72%
700samps_a*	**0.673**	**59.37%**	0.840	76.69%
1000samps_o	0.951	**86.17%**		
1000samps_s	0.972	91.60%	**0.927**	92.69%
1000samps_a	0.955	**85.67%**	0.988	98.81%
1000samps_a*	**0.934**	**83.30%**	**0.934**	93.38%
100samps_o	0.894	84.69%		
100samps_s	0.889	89.80%	0.831	83.67%
100samps_a	**0.853**	**77.55%**	**0.824**	82.65%
100samps_a*	**0.723**	78.57%	0.767	77.55%

[a]Data set identifier used to distinguish each data set where the numeric value is the rounded training samples and the letter is the feature trimming method (o = original data set, s = significance feature selection, a = modestly trimmed abundance feature selection, and a* = heavily trimmed abundance feature selection). [b]SVM could not perform the classifications on the original data sets due to memory limitations, these missing values are highlighted with a red box. Values in boldface are at least 5% lower than the respective results for XGBoost.
